# Long term health consequences of a career in professional horse racing: the prevalence of pain amongst retired race jockeys

**DOI:** 10.1186/1753-6561-6-S4-O53

**Published:** 2012-07-09

**Authors:** A Tomkinson, H Watts, AL Mackinnon, RJ O'Connor

**Affiliations:** 1 University of Leeds, UK; 2 Professional Jockey's Association; 3 Academic Department of Rehabilitation Medicine, Leeds Institute of Molecular Medicine, University of Leeds, Leeds, UK

## Introduction

Horse racing is an exhilarating and high-risk sport. Over 500 jockeys were injured in the UK in the last two decades. Jockeys have racing careers lasting up to 40 years, yet little is known about the long-term consequences of multiple falls and the consequent impact on health. The aim of this study is to establish the impact of musculoskeletal injuries in retired jockeys.

## Methods

A prospective, cross-sectional survey of retired jockeys registered with the Professional Jockey Association using a questionnaire to examine current musculoskeletal pain (measured using a numerical rating scale) in nine key anatomical areas, previous injuries and treatments for injuries.

## Results

One hundred and twenty retired jockeys returned questionnaires; 90% had experienced a musculoskeletal injury. Mean total pain at rest was 11. There is a significantly greater total score for pain with movement (13; p < 0.05). Pain in the lower back was most common. Eighty percent of respondents believed the pain they experience now is as a result of the injuries they sustained during their racing career; 22 respondents' careers ended due to one or more musculoskeletal injury.

## Conclusions

There is a higher prevalence of pain in this sample population than the general population. The most common area for pain was the lower back and this is in common with the findings of studies of the general population and in retirees of other sports. This clinical relevance of these findings is that there is an occupational health hazard of a career in race riding as it can lead to pain in later life.

